# A Novel Distributed Hybrid Cognitive Strategy for Odor Source Location in Turbulent and Sparse Environment

**DOI:** 10.3390/e27080826

**Published:** 2025-08-04

**Authors:** Yingmiao Jia, Shurui Fan, Weijia Cui, Chengliang Di, Yafeng Hao

**Affiliations:** 1 School of Electronic and Information Engineering, Hebei University of Technology, Tianjin 300401, China; 202211901001@stu.hebut.edu.cn; 2 Innovation and Research Institute, Hebei University of Technology, Shijiazhuang 050299, China; 3 The 54th Research Institute of China Electronics Technology Group Corporation, Shijiazhuang 050081, China; cwjbuaa@126.com (W.C.); dichengliang@163.com (C.D.); datoushr@126.com (Y.H.)

**Keywords:** odor source localization, Bayesian inference, Rényi entropy, particle filter, gravitational potential field, sequential Monte Carlo, autonomous search

## Abstract

Precise odor source localization in turbulent and sparse environments plays a vital role in enabling robotic systems for hazardous chemical monitoring and effective disaster response. To address this, we propose Cooperative Gravitational-Rényi Infotaxis (CGRInfotaxis), a distributed decision-optimization framework that combines multi-agent collaboration with hybrid cognitive strategy to improve search efficiency and robustness. The method integrates a gravitational potential field for rapid source convergence and Rényi divergence-based probabilistic exploration to handle sparse detections, dynamically balanced via a regulation factor. Particle filtering optimizes posterior probability estimation to autonomously refine search areas while preserving computational efficiency, alongside a distributed interactive-optimization mechanism for real-time decision updates through agent cooperation. The algorithm’s performance is evaluated in scenarios with fixed and randomized odor source locations, as well as with varying numbers of agents. Results demonstrate that CGRInfotaxis achieves a near-100% success rate with high consistency across diverse conditions, outperforming existing methods in stability and adaptability. Increasing the number of agents further enhances search efficiency without compromising reliability. These findings suggest that CGRInfotaxis significantly advances multi-agent odor source localization in turbulent, sparse environments, offering practical utility for real-world applications.

## 1. Introduction

The leakage of toxic, harmful, or combustible gases poses a serious threat to the safety of the general public. Odor Source Localization (OSL) is a pivotal research domain within the fields of robotics and environmental monitoring [1]. Its objective is to develop methods and technologies capable of swiftly and accurately determining the location of odor sources through the concentration reading of pollutants (or harmful substances) in the air. Odor source location research has broad application prospects in detecting air pollution [2], preventing and mitigating dangerous gas leakage disasters [3], and rescuing survivors in damaged buildings [4].

Early fixed (wireless) sensor networks are widely utilized in environmental monitoring [5]. However, most dangerous gas leaks occur suddenly, and thus the location of the leak is somewhat random, posing challenges in pre-deploying fixed sensor network nodes in unknown location [6]. Moreover, sustaining high-density fixed sensor networks over extended periods is impractical. With advancements in science and technology, researchers have proposed numerous strategies for estimating odor sources using mobile sensors, such as drones [7], rather than fixed sensor networks. Mobile sensors offer the advantage of swiftly covering large and diverse areas with minimal sensors. Mobile robots equipped with odor sensors detect target odors across multiple locations, adjust their path and direction via path planning or decision-making processes [8], and ultimately locate the odor source.

The methods used to complete the OSL tasks can be generally divided into three groups [9]: bioinspired, concentration gradient-climbing, and probabilistic algorithms. Bioinspired strategies mimic the olfactory behavior of animals or insects to find food [10]. Typical algorithms include the moth heuristic algorithm [10,11], the particle swarm optimization algorithm [12], the whale optimization algorithm [13], etc. Some researchers [14] have also applied bionic strategies to multi-agent systems to facilitate information sharing and mutual cooperation among agents.

The concentration gradient-climbing algorithm calculates the local concentration gradient of a gas plume and moves the mobile sensor to a higher concentration. These strategies are simple and effective under well-defined conditions but are less effective due to the sparse, intermittent concentration tracks formed by odor plumes in turbulent environments. The probabilistic algorithm uses Bayesian inference [15] to search for odor sources [16]. Instead of directly following odor plumes according to reliable sensor measurements, the probabilistic algorithm makes sparse measurements in areas that may reduce the uncertainty of source location, so it is more suitable for odor source location research in a turbulent environment. The infotaxis algorithm [17], proposed by Vergassola et al. in 2007, is one of the earliest probabilistic algorithms.

However, in addressing disasters and emergencies, the primary task of locating odor sources is to ensure speed and safety. Multi-agent systems [18] offer advantages in quickly searching for sources and accurately estimating them by acquiring more information. Consequently, the collaborative search using multi-agent systems has attracted researchers’ attention. In 2017, Vadas Gintautas et al. [19] first proposed the idea of synergy to improve the infotaxis performance of multiple searchers, defining the search problem for multiple agents based on infotaxis.

The application of infotaxis in [17,20,21] exhibits inherent limitations in localizing odor sources. The algorithm relies on a grid-based source probability map for its estimation and decision-making processes. The search space is discretized into a two-dimensional grid with uniformly scaled probability values. These values indicate the likelihood of odor plume presence in each grid cell, aiding in path planning. Yet, higher spatial resolution necessitates smaller grid sizes, increasing computational complexity. Optimal source probability approximation requires a sufficiently dense grid, but this increases computational overhead. Bayesian estimates suffer from the curse of dimensionality. To address this issue, the particle filter method [22] is introduced to enhance the accuracy of probability map approximation and Bayesian sequential estimation in infotaxis.

It is worth noting that the probabilistic framework for diffusion source localization is not limited to odor tracking but also holds significant relevance in networked domains such as epidemic propagation and information dissemination. Yu et al. systematically elaborated on the mathematical foundations and algorithmic designs for source detection in large-scale networks [23], highlighting that incorporating prior knowledge of network topology can substantially enhance detection performance, especially under limited local observations. Notably, the optimization task of identifying “superspreaders” in social networks exhibits a structural analogy to the collaborative localization of high-probability odor sources by multi-robot systems in turbulent environments, particularly in terms of decision-making objectives.

With these backgrounds, this paper proposes a hybrid cognitive strategy for odor source localization based on the Monte Carlo framework. The primary contributions of this approach are as follows:

(1) **Hybrid Cognitive Strategy for Turbulent Environment Navigation:** The paper develops a decision architecture synergizing gravitational potential fields with Rényi entropy optimization, specifically engineered for odor source localization in atmospheric turbulence. The entropy module quantifies uncertainty in sparse concentration fields and the gravitational drives the rapid convergence for the multi-agent.
(2) **Distributed Interactive Optimization for Collaborative Pollution Monitoring:** The distributed decision-making framework implements a dynamic consensus protocol enabling multi-agent coordination in sparse environments. Decentralized coordination minimizes sensor processing demands while accelerating adaptation to atmospheric dispersion patterns.
(3) **Particle Filtering for Sparse Signal Processing:** Particle Filter is introduced, using randomly sampled particles to characterize the source probability distribution in the environmental space. The particle filter can adaptively distribute particles in the target area based on observations, enhancing localization accuracy and computational efficiency.

The rest of this paper is arranged as follows: The related work for OSL will be discussed in Section 2, which highlights important design gaps in the existing literature. The problem formulation is presented in Section 3, encompassing the gas dispersion model and the sensor measurement model. Section 4 introduces a new hybrid strategy based on a Monte Carlo framework, which integrates Bayesian estimation, particle filter sampling, a source confirmation method, a distributed interactive decision-optimization method, and an autonomous agent decision-making strategy. In Section 5, various algorithms are simulated within a virtual environment, including parameter selection, illustrative runs, and Monte Carlo simulations. Finally, conclusions and directions for future work are provided in Section 6.

## 2. Related Work

After the emergence of infotaxis, many researchers began adopting probabilistic algorithms to search for unknown odor sources. Infotaxis introduces Bayesian inference into the odor source localization problem, with the objective of estimating the probability distribution of the source parameters rather than directly identifying an optimal solution. This approach involves evaluating the posterior probability distributions of the parameters through random sampling, enabling a comprehensive characterization of both the source parameters and their associated uncertainty. This also inspired researchers to develop a solution for odor source localization that does not rely on concentration gradients. Since then, researchers have continued to extend and experiment with the infotaxis approach. Masson et al. [24] demonstrated the effectiveness of infotaxis in a single odor source environment through quantitative tests, evaluating its robustness relative to inappropriate transport models. Moraud et al. [25] evaluated the performance of information directivity under sparse conditions through a combination of robot experiments and simulations. Eggels et al. [26] tested the infotaxis algorithm in a number of simulated 3D turbulent channels. Ruddick et al. [27] extended the application range of infotaxis to three dimensions, addressing the problem of locating odor leakage sources in three-dimensional room space.

In the infotaxis algorithm [17], the agent has five possible actions for forming a square grid, where the reward function is set according to the expected change in entropy and moves towards the direction of the maximum expected change in entropy. Recent advancements in cognitive search strategies have introduced new approaches. Ristic et al. suggested that entropy reduction when finding the source makes sense only if the source location coincides with one of the nodes of the square lattice. As a response, they propose the infotaxis II search strategy [28], which omits the “exploitation” component from the reward function. Michael et al. [29] presented an alternative approach wherein the search was guided by the maximum entropy sampling principle. This strategy was tested in 2019 using a mobile robotic device [30]. Faezeh et al. [31] derived a novel reward function using weighted KL divergence and information entropy, successfully implementing experiments with the Khepera IV robot. Zhu, Hongbiao et al. [32] introduces an artificial potential field algorithm into infotaxis to mitigate the influence of pseudo-sources caused by obstacles. Zhao et al. [33] employed cognitive strategies in obstacle-laden environments, combining Infotaxis and entrotaxis algorithms with a passive escape mechanism, as verified through simulations using Fluent software. Hernandez-Reyes et al. [34] fused infotaxis with a silk moth-mimetic algorithm, observing enhanced performance with larger agent sizes. Loisy et al. [20] combined spatial information with infotaxis, proposing Space-Aware Infotaxis (SAI) and demonstrating its reliability under high source densities. Luong et al. [4] integrated the infotaxis algorithm with Dijkstra algorithm to achieve odor source localization in multi-obstacle environments. Jiu et al. [35] introduced a path planning strategy based on a partially observable Markov decision process algorithm and an artificial potential field algorithm. Liu et al. [21] devised the adaptive space-aware infotaxis II algorithm to solve the problem of low search efficiency and local optimality of existing odor source localization strategies. Zhao et al. [36] proposed a method that uses regression techniques to predict gas concentrations and entrotaxis to determine the location of the odor source, thereby conducting source search.

The extended application of the infotaxis algorithm in multi-agent systems has been a focal point of research in the field of odor source localization. In sensor and robot networks, the global estimation of the belief distribution of the source can be conducted through either centralized or distributed approaches. Compared to centralized estimation, distributed estimators offer greater robustness and scalability in estimating global posterior beliefs, likelihood functions, and other statistics, making them well-suited for large-scale robot networks. Additionally, the distributed approach significantly reduces the computational load, thus facilitating deployment in real-world scenarios. In 2017, Karpas et al. [37] combined infotaxis with social interaction, introducing the “socialtaxis” algorithm to facilitate odor source localization in a collective context. In 2020, Park et al. [38] proposed three coordination methods based on infotaxis, demonstrating the advantages of collaborative systems. In 2022, Ji et al. [39] combined the entrotaxis algorithm with an intermittent search algorithm in the field of multi-robot collaboration, introducing a new search algorithm, the Entrotaxis-Turn (ET) algorithm, which demonstrated superior performance in environments with obstacles. Duan et al. [40] proposed an automatic drive model based on game theory and reinforcement learning, which enables multi-agents to carry out strategic reasoning with negotiation in traffic scenarios. In 2023, Wang et al. [41] constructed a multi-agent massive target cooperative search mission planning model based on a reinforcement learning algorithm.

The introduction of the particle filtering algorithm into the infotaxis framework represents a significant advancement in probabilistic search strategies by effectively mitigating the curse of dimensionality. Infotaxis II [28] and entrotaxis [29] integrate the particle filter into traditional infotaxis, employing Monte Carlo sampling to approximate the probability map for Bayesian estimation. Park et al. [38,42] integrated infotaxis with a Gaussian mixture model, which clusters potential source locations identified by the particle filter to predict the next probable source location. Ristic et al. [43] utilized the rao-blackwell particle filter to achieve autonomous search in an environment with an unknown map of randomly placed and shaped obstacles. Jang et al. [9] enhanced socialtaxis by implementing a Rao-Blackwellized particle filter to transition from a gridded to a continuous environment, alongside introducing a novel reward function. Nanacati et al. [44] integrates distributed Bayesian filtering, coverage control, information-theoretic sampling, and proximity constraint handling, forming an efficient and fully distributed coordination protocol.

Source confirmation represents the final stage of odor source localization, where a rational approach to confirmation can attenuate the impact of pseudo-sources, thereby elevating the success rate and precision of localization. Zhu et al. [32] introduced a statistical-based source confirmation method aimed at filtering out pseudo-source and ensuring accurate target localization. In refs [20,45], a thresholding approach was employed; upon reaching the threshold, the presence of the odor source was confirmed. Wang et al. [46] suggested utilizing external sensors, such as cameras, for practical source confirmation. Other researchers [27,47] had proposed diverse termination criteria for algorithms to comprehensively ascertain the presence of an odor source, considering factors like entropy and agent location.

Based on previous studies, hybrid cognitive strategies are proposed for multi-agent systems by integrating a gravitational potential field related to spatial distance into a probabilistic framework. Additionally, λ is introduced to dynamically regulate the balance between exploration and exploitation, complemented by a distributed interaction optimization strategy and a source determination method to enable the rapid localization of odor sources in turbulent and sparse environments.

## 3. Problem Description

The paper investigates the application of mobile robots for odor source localization. The task of mobile robot odor source localization entails directing the robot to autonomously locate and reconstruct the odor source. The mobile robot is equipped with mobility and a flexible configuration, enabling it to be fitted with various gas sensors or electronic nose systems tailored to specific odor sources, thereby enhancing its safety and adaptability.

The paper assumes the position of an unknown odor source, denoted as $r_{s}$, and employs a gas diffusion model to characterize its properties. The sensor is affixed to the mobile robot. At time *k* (during which the mobile robot moves one step per second), the sensor’s position is denoted as $r_{k}$, and it measures the ambient concentration. In this context, the sensor functions as a node within the sensor network. Its measurements are employed in complex reasoning calculations to ascertain the subsequent movement direction.

Previous studies have modeled odor propagation, concentration distribution, and sensor perception as mathematical functions, utilizing probabilistic reasoning to infer potential locations of odor sources. This approach facilitated the resolution of the actual problem of odor source localization, thereby enhancing the interpretability and efficiency of the solution. The mathematical formulas related to odor diffusion and sensor perception, including the gas diffusion model and the sensor measurement model, will be elucidated in subsequent sections.

### 3.1. Gas Dispersion Model

The model of particle emission into a turbulent atmosphere is employed as the dispersion model in this study [17] and is called the isotropic turbulence model. This model is suitable for gases that diffuse within a carrier fluid characterized by sparse and irregular traces, such as toxic gases and pollen. In this framework, detectable “odor particles” are emitted from a source located at $r_{s}$ with a release rate of $Q_{s}$. The model assumes that emitted particles possess finite lifetime τ and propagate with isotropic diffusivity *D*. The average wind speed in the environment is denoted as *V*, with its direction along the positive half-axis of the X-axis.

The mean stationary concentration field $c(r|r_{s})$ generated by a source at position $r_{s}=\left[x_{s},y_{s}\right]$ satisfies the following advection-diffusion equation.

(1) $$0=V{▿}_{x}c\left(r|r_{s}\right)+D\Delta c\left(r|r_{s}\right)-\frac{1}{\tau }+Q_{s}\delta \left(r-r_{s}\right)$$

**Figure 1:**
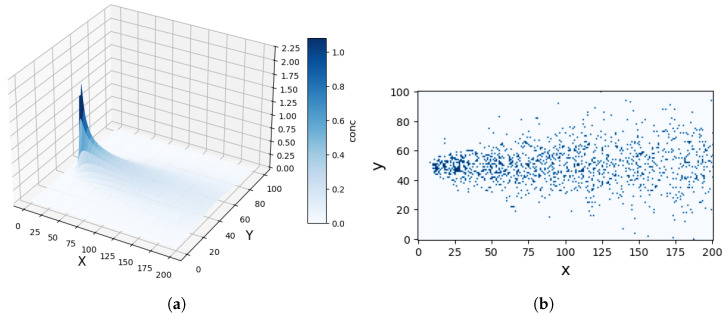
Isotropic turbulence model, a=1, $r_{s}=\left[10,50\right]$, $Q_{s}=1$, D=1, V=1, τ=400. (**a**) $c(r|r_{s})$ in 2D scenario with sensing size. (**b**) Sensor measurement in 2D scenario.

It should be noted that, in studying decision-making and information sharing among multiple agents, the isotropic turbulence models employed in this study assumed steady wind conditions (average wind speed *V*), neglecting temporal variations in both wind speed and direction. This assumption facilitated a focus on the algorithmic framework but limited the models’ capability to capture the temporal dynamics of outdoor plumes. Consequently, realistic validation under dynamic wind conditions is identified as a critical direction for future research. In 2D, the solution reads:

(2) $$c\left(r|r_{s}\right)=\frac{Q_{s}}{2\pi D}exp\left(\frac{(x-x_{s})V}{2D}\right)K_{0}\left(\frac{|r-r_{s}|}{\lambda }\right)$$

(3) $$\lambda =\sqrt{\frac{D\tau }{1+\frac{V^{2}\tau }{4D}}}$$

where λ represents the correlation length of the source and $K_{0}$ is the modified Bessel function of order zero. Figure 1a depicts the gas concentration distribution in a two-dimensional environment, highlighting the significant challenges posed by sparse and turbulent conditions in terms of source localization.

### 3.2. Sensor Measurement Model

The gas diffusion behavior of the odor source is modeled by Equation (2). Assuming the sensor has a search diameter of *a*, gas detection can be regarded as a spherical searcher with diameter *a*. The sensor, positioned at $r_{k}$, interacts with diffused gas particles in the environment, resulting in a series of encounters that occur with a certain frequency:

(4) $$R\left(r_{k}|r_{s}\right)=\frac{2\pi Dc(r_{k}|r_{s})}{ln(\frac{\lambda }{a})}=\frac{Q_{s}}{ln(\frac{\lambda }{a})}exp\left(\frac{(x_{k}-x_{s})V}{2D}\right)K_{0}\left(\frac{|r_{k}-r_{s}|}{\lambda }\right)$$

At time *k*, the number of particles detected by the sensor at position $r_{k}$ is influenced by the environment and the type of sensor. To simplify the sensor measurement model, particle detection behavior is categorized into *F* (indicating particles detected, non-zero measurements) and $\bar{F}$ (indicating no particles detected, zero measurements). Sensor detection is modeled as a stochastic process, where the interaction between the sensor and the emitted particles is represented by a Poisson distribution: the probability of a sensor positioned at $r_{k}$ encountering a particle within the sampling time Δt can be expressed as follows:

(5) $$p\left(z_{k}|\mu_{k}\right)=\frac{{\mu_{k}}^{z_{k}}}{z_{k}!}exp\left(-\mu_{k}\right)$$

where $\mu_{k}=R\left(r_{k}|r_{s}\right)*\Delta t$ represents the average number of particles detected during the time interval Δt and $z_{k}$ represents the number of particle detections. For the sake of simplicity in calculations, detection occurs in the *F* case when $z_{k}>0$, and non-detection in the $\bar{F}$ case when $z_{k}=0$. Therefore, the equation can be expressed as:

(6) $$p\left(\mu_{k}\right)=\left\{\begin{array}{c} 1-exp(-\mu_{k}),F\\ exp\left(-\mu_{k}\right),\bar{F} \end{array}\right.$$

Figure 1b illustrates the observations of a searcher at a fixed point in time. It is observed that particles exhibit positional discontinuity, indicating that the gradient method cannot be utilized to locate the odor source in a sparse environment.

## 4. Source Location Method

This section outlines the process through which the agent detects the odor plume as a cue to approach the odor source and collects sufficient information to ascertain its location, thereby completing the OSL task. This paper employs real-time measured information to perform Bayesian inference and update the probability map. The CGRInfotaxis method is proposed for determining movement direction, while an interactive decision optimization approach is employed to achieve dynamic decision updates and information sharing. Finally, a specialized algorithm is implemented to accurately identify the odor source, enabling cooperative odor source localization among multiple agents.

### 4.1. Bayesian Estimation

The Bayesian framework [48] is employed to estimate the source position based on uncertain information. In this study, to streamline calculations and rigorously evaluate algorithm performance, it is assumed that various parameters required by the dispersion model in Equation (2)—such as wind speed *V*, diffusion coefficient *D*, and particle survival time τ—are known. At time *k*, the agent moves to position $r_{k}=\left[x_{k},y_{k}\right]$, where the measured value is $z_{k}$ and the predicted source parameters are $\theta_{k}$. Thus, the posterior probability distribution function for the estimated source term is represented by the prior probability function and the sensor measurement, according to Bayes’ theorem.

(7) $$p\left(\theta_{k}|z_{1:k}\right)=\frac{p\left(z_{k}|\theta_{k}\right)p\left(\theta_{k}|z_{1:k-1}\right)}{p(z_{k}|z_{1:k-1})}$$

where $p\left(z_{k}|z_{1:k-1}\right)=\int p\left(z_{k}|\theta_{k}\right)p\left(\theta_{k}|z_{1:k-1}\right)d\theta_{k}$ denotes the normalized constant and $p(z_{k}|\theta_{k})$ represents the likelihood function, indicating the probability distribution of observing $z_{k}$ at position $r_{k}$ given the source parameters $\theta_{k}$. $p(\theta_{k}|z_{1:k-1})$ signifies the prior probability at time k, which equivalently serves as the posterior probability at time k−1.

If prior information about the source is available before the search, it can be represented by an appropriate distribution. However, in the absence of this information, the initial prior distribution is set to a uniform distribution $p\left(\theta_{0}\right)=U\left(\Omega \right)$. In this study, a uniform distribution over the domain Ω is employed. In subsequent iterations, the probability distribution is updated using the likelihood function until the odor source is identified.

In Equation (7), the likelihood function represents the probability distribution of whether or not position $r_{k}$ receives non-zero measurements given the source parameter $\theta_{k}$. The sensor measurement model is formulated using the Poisson distribution. The probability of encountering particles released by source $\theta_{k}$ at position $r_{k}$ in unit time is given by Equation (5). Substituting $\mu_{k}=R\left(z_{k}|\theta_{k}\right)*\Delta t$ into Equation (6) yields the equation below, where $R(z_{k}|\theta_{k})$ is derived from Equation (4).

(8) $$p\left(z_{k}|\theta_{k}\right)=\left\{\begin{array}{c} 1-exp(-R\left(z_{k}|\theta_{k}\right)*\Delta t),F\\ exp\left(-R\left(z_{k}|\theta_{k}\right)*\Delta t\right),\bar{F} \end{array}\right.$$

### 4.2. Particle Filter

Equation (7) involves a posterior probability distribution that is challenging to compute analytically due to the presence of continuous integrals in the denominator. Additionally, employing the raster probability map method significantly increases the computational complexity of the algorithm. Particle Filters (PFs) are utilized to perform Bayesian estimation of source parameters within the sequential Monte Carlo framework. The fundamental concept of PFs is to approximate the posterior probability distribution $p(\theta_{k}|z_{1:k})$ using a set of randomly sampled particles with associated weights. The integral in Equation (7) can be approximated using the posterior distribution of weighted particles. This will be referred to as a potential source term to distinguish it from particles emitted by the source and those used in the particle filter.

Given the weighted samples $\theta_{k}^{\left(m\right)}=\left\{r_{k}^{\left(m\right)},w_{k}^{\left(m\right)}\right\},m=1,2,\ldots M$, the posterior distribution in Equation (7) can be approximated as follows:

(9) $$p\left(\theta_{k}|z_{1:k}\right)\approx \sum_{m=1}^{M}w_{k}^{\left(m\right)}\delta \left(r-r_{k}^{\left(m\right)}\right)$$

where $r_{k}^{\left(m\right)}$ denotes the position of the *m*-th potential source term, $w_{k}^{\left(m\right)}$ represents its normalized weight, and $\sum_{m=1}^{M}w_{k}^{\left(m\right)}=1$. Here, δ(·) denotes the Dirac delta function. Direct sampling from the posterior probability $p(\theta_{k}|z_{1:k})$ is challenging. Therefore, the Sequential Importance Sampling (SIS) technique is employed. Samples are drawn from $q(\theta_{k}|z_{1:k})$, which is easier to sample from and is referred to as the proposal distribution.

The weights are updated using sequential importance sampling, while the position distribution of particles remains unchanged, with only the particle weights being updated. An approximation $p(\theta_{k}|z_{1:k})$ of the new particle $\theta_{k}^{\left(m\right)}$ is obtained through $q(\theta_{k}^{\left(m\right)})$. The corresponding sample weights are subsequently updated according to:

(10) $${\bar{w}}_{k}^{\left(m\right)}=w_{k-1}^{\left(m\right)}\frac{p\left(\theta_{k}^{\left(m\right)}|\theta_{k-1}^{\left(m\right)}\right)p\left(z_{k}|\theta_{k}^{\left(m\right)}\right)}{q(\theta_{k}^{\left(m\right)}|\theta_{k-1}^{\left(m\right)},z_{k})}$$

In this study, it is assumed that the odor source is time-invariant, meaning that its location and release rate do not change over time. This leads to the equation where $\theta_{k}^{\left(m\right)}=\theta_{k-1}^{\left(m\right)}$ for m=1,2,…M. We assume that the suggestion distribution is a posterior probability distribution at time k−1, i.e., $q\left(\theta_{k}^{\left(m\right)}\right)=p\left(\theta_{k-1}|z_{1:k-1}\right)$. The normalized particle weights are updated accordingly:

(11) $${\bar{w}}_{k}^{\left(m\right)}=w_{k-1}^{\left(m\right)}p\left(z_{k}|\theta_{k}^{\left(m\right)}\right)$$

The normalized weight is easily computed as

(12) $$w_{k}^{\left(m\right)}=\frac{{\bar{w}}_{k}^{\left(m\right)}}{\sum_{m=1}^{M}{\bar{w}}_{k}^{\left(m\right)}}$$

To prevent particle degradation, resampling is necessary when the effective particle number falls below the set threshold $M_{th}$. The effective particle number $M_{eff}$ is calculated as follows:

(13) $$M_{eff}=\frac{1}{\sum_{m=1}^{M}{\left(w_{k}^{\left(m\right)}\right)}^{2}}$$

When $M_{eff}<M_{th}$, particles are resampled, redistributing their positions in space, and their weights are reset to $\frac{1}{M}$. When the resampling condition is not met, particle positions remain unchanged, and their weights are sequentially updated according to Equation (11).

### 4.3. Source Confirmation Method

**Figure 2:**
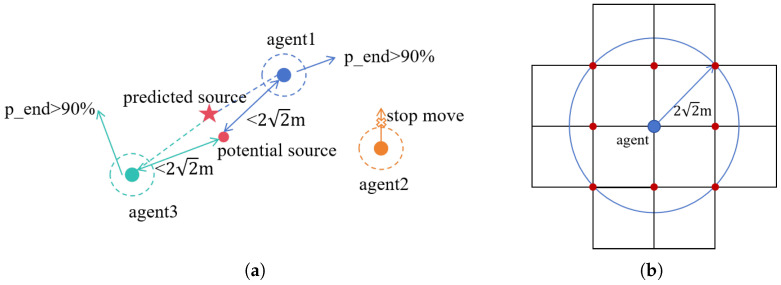
Source confirmation method. (**a**) Illustration. (**b**) Threshold selection.

Source confirmation represents the final stage of the odor source localization algorithm. Typically, in nature, organisms confirm the source using their sensory capabilities, such as vision and touch, upon reaching the vicinity of the source. However, agents may lack such sensors and cannot depend on biological sensory systems for precise source confirmation. Therefore, an appropriate method for source confirmation is essential. This paper mainly relies on the detected probability of the source and the distance to potential sources as the primary basis for detecting the odor source, as shown in Figure 2a. In the odor source search process, if the following conditions are met, the algorithm terminates, and the agent’s current position is considered the odor source location.

(1) $p_{end}>90\%$. Here, $p_{end}$ represents the probability that the agent’s current location is the source. Based on the distribution of highly weighted particles around the agent, when these particles are densely concentrated, it is concluded that the agent is near the odor source.
(2) The distance between the potential sources and the agent is less than $2\sqrt{2}$ m. In this study, a series of weighted particles is used to approximate the posterior probability distribution. The agent moves towards the potential source with higher probability. When the distance between the high-probability sources and the agent is less than $2\sqrt{2}$ m, it is inferred that the agent is likely to be near the odor source.

Since the agent moves with a fixed step size of 2 m, its movement is restricted to discrete grid nodes, and the agent is considered likely to have located the odor source when the position of the potential source center satisfies the “neighboring” condition relative to the agent. Accordingly, the distance threshold is defined as the maximum distance of $2\sqrt{2}$ m within the neighborhood, as illustrated in Figure 2b. The blue node indicates the agent’s location, while the red nodes represent all adjacent grid nodes. If the Euclidean distance between the potential source center and the agent is less than or equal to this threshold, the potential source is considered to reside within the agent’s neighboring grids, thereby satisfying the “neighboring” condition.

In the agent search process, the odor source is considered found only when both of the above conditions are simultaneously satisfied. In the collaborative localization of odor sources by multi-agents, we define that if any single agent meets the specified conditions, it is considered to have located the odor source. During the search process, if any agent identifies the odor source, it is assumed that the source has been confirmed, prompting all agents to cease movement. The predicted source location is taken as the position predicted by the agent that found the source. If multi-agents simultaneously identify the odor source, the predicted source location is considered to be the centroid of the expected sources identified by the agents. Figure 2a shows the process of odor source confirmation by three agents; agent1 and agent3 find the odor source, and the agents stop searching at the same time. The predicted source that the two agents finally confirmed is represented by a red five-pointed star.

### 4.4. Distributed Interactive Decision-Optimization Method

**Figure 3:**
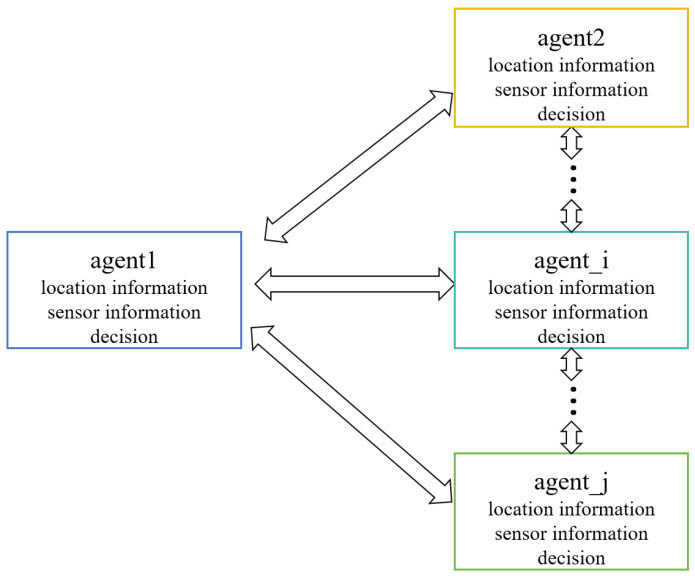
Information sharing mechanism between agents.

In the multi-agent system, each agent must work collaboratively based on local and global information. In multi-agent collaborative tasks, the decision-making of a single agent is influenced not only by its own sensors but also by the actions of other agents. The information sharing mechanism between agents is shown in Figure 3. Information is exchanged between each agent. Therefore, this paper proposes a distributed interactive decision optimization method, which achieves global optimality through interaction and updates based on information exchange and joint decision-making between agents.

**Figure 4:**
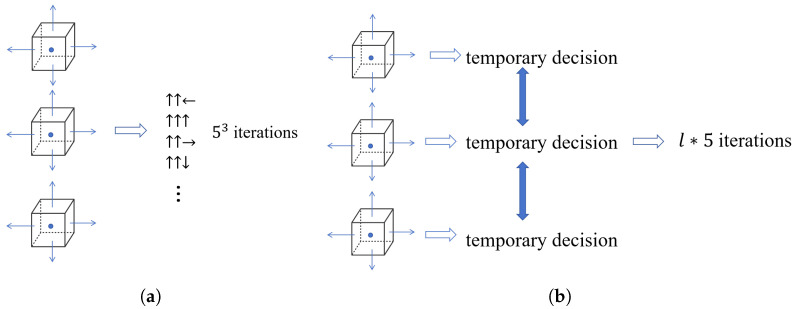
Decision computation (three agents as an example). (**a**) Centralized decision. (**b**) Distributed interactive decision.

**Figure 5:**
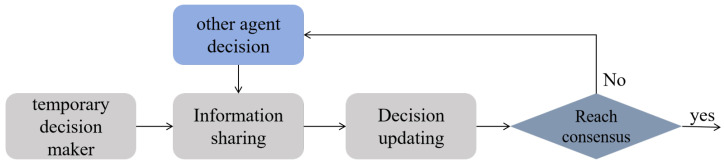
Framework of distributed interactive decision optimization method.

For optimization methods involving multiple agents, the most reliable approach involves iterating through each agent’s possible actions to find the optimal direction. However, this method has a huge computational cost. Supposing we use *N* agents to search the source in a 2D space, each decision requires $5^{N}$ iterations. As shown in Figure 4a, taking three agents as an example, it need 125 iterations in total to find the optimal direction. When the number of agents is large, the computation speed becomes very slow, resulting in computation speeds that fail to meet real-time requirements. However, the distributed interactive decision-optimization method only needs l∗5 iterations to find the optimal direction, as shown in Figure 4b. *l* represented the number of negotiation times between agents. The main framework of the distributed interactive decision-optimization method is shown in Figure 5.

The core idea of the interactive decision-making method is that after each agent makes its initial temporary decision, it gradually optimizes its decision based on communication with other agents. As shown in the figure, this process includes the following steps:

(1) Temporary decision maker: Each agent generates an independent temporary decision based on its own sensor information. This decision depends on factors such as the gas concentration perceived by the agent, the current probability map information, and the current position.
(2) Information sharing: After making the temporary decision, each agent shares it with others through a network. The shared information includes the agent’s decision as well as the current sensor measurements.
(3) Decision update: Based on the decisions received from other agents and the gas perception results, each agent adjusts its decision by integrating its own sensory data. This process is based on the proposed CGRInfotaxis algorithm, which will be described in detail in Section 4.5.
(4) Reach consensus: After several rounds of information exchange and decision updates, all agents ultimately form a unified decision. The behaviors of the agents converge, effectively avoiding resource waste or unnecessary conflicts caused by decision divergences.

### 4.5. Decision Making for Autonomous Agent

The cognitive strategy directs the agent to move in a predefined direction with a fixed step size. The optional action set A contains 5 actions, i.e., A=[↑,↓,←,→,·], where the symbols denote movements: ↑ for forward, ↓ for backward, ← for leftward, → for rightward, and · for staying in the original position. Assume the agent moves with a step size Δstep. In the source confirmation method (Section 4.3), a fixed movement step length of 2 m was set, and the distance threshold conditions were calculated based on this value. This step length is aligned with the Δstep parameter employed throughout the decision-making process. In this study, Δstep was uniformly defined as 2 m. At time k+1, the agent’s position $r_{k+1}=\left[x_{k+1},y_{k+1}\right]$ is calculated as follows. Note that although Δstep is set to 2 m, agents may also remain stationary in certain situations, corresponding to a step size of 0.

(14) $$\left\{\begin{array}{c} x_{k+1}=x_{k}+\Delta step\\ y_{k+1}=y_{k}+\Delta step \end{array}\right.$$

At time *k*, suppose the agent moves according to the optional action set $A_{k}$, where $A_{k}=A$. The agent’s optional movement positions are denoted by $a_{k}$. In an odor source search using a multi-agent system, each agent moves according to its optional action set. Therefore, the collective action set, formed by combining the actions of multiple agents, is defined as $A_{ck}$, and the collective movable positions are determined as $a_{ck}\in A_{ck}$.

In multi-agent motion scenarios, collision detection is essential to ensure safe and efficient navigation. A collision is considered to occur if the distance between any two agents is less than 1 m. When such collisions are detected, the corresponding positions of the agents involved are removed to maintain operational integrity and avoid conflicts in the system.

#### 4.5.1. Classical Cognitive Strategy

When the agent moves based on the optional action set, it uses its sensors to gather useful information about its surroundings. In the classic infotaxis algorithm [17], Shannon entropy reduction is employed to represent the information state of the agent concerning the source’s location, thereby quantifying the source’s uncertainty. The core concept involves selecting the next optimal action based on Markov decision processes. As the decision criterion for the robot, the information entropy reduction must clearly demonstrate the advantages and disadvantages of alternative movement directions to facilitate the optimal choice. The formula is provided below:

(15) $$I_{info}\left(a_{k}\right)=p\left(r_{k+1}\right)\left[-H_{k}\right]+\left(1-p\left(r_{k+1}\right)\right)\left[E\left({\hat{H}}_{k+1}\left({\hat{z}}_{k+1}\right)\right)-H_{k}\right]$$

In Equation (15), the first term is relevant only when the source location $r_{s}$ coincides with the $r_{k+1}$, so it is discarded. The infotaxis II reward [28], denoted as $I_{info2}\left(a_{k}\right)$, is then solely represented by the second term in Equation (15) when $p(e_{k+1}=0)$, which is

(16) $$I_{info2}\left(a_{k}\right)=E\left({\hat{H}}_{k+1}\left({\hat{z}}_{k+1}\right)\right)-H_{k}$$

where $H_{k}$ denotes the Shannon entropy at time *k*, $E({\hat{H}}_{k+1}\left({\hat{z}}_{k+1}\right))$ represents the expected entropy at time k+1, and ${\hat{z}}_{k+1}$ denotes the hit value at time k+1, with its probability distribution following a Poisson distribution as described in Section 4.1. ${\hat{H}}_{k+1}$ represents the entropy at time k+1, given by Equation (17).

(17) $${\hat{H}}_{k+1}=-\int p\left({\hat{\theta }}_{k+1}|{\hat{z}}_{1:k+1}\right)logp\left({\hat{\theta }}_{k+1}|{\hat{z}}_{1:k+1}\right)d{\hat{\theta }}_{k+1}$$

where $p({\hat{\theta }}_{k+1}|{\hat{z}}_{1:k+1})$ can be approximated by a particle filter. According to Equation (11), the non-normalized weight of the potential source term at k+1 time can be update as:

(18) $${w^{\prime }}_{k+1}^{\left(m\right)}=p\left({\hat{z}}_{k+1}|\theta_{k+1}^{\left(m\right)}\right)*w_{k}^{\left(m\right)}$$

$w_{k+1}^{\left(m\right)}=\frac{{w^{\prime }}_{k+1}^{\left(m\right)}}{\sum_{i=1}^{M}{w^{\prime }}_{k+1}^{\left(m\right)}}$ is a normalized weight, and the Shannon entropy at k+1 is:

(19) $${\hat{H}}_{k+1}=-\sum_{m=1}^{M}w_{k+1}^{\left(m\right)}logw_{k+1}^{\left(m\right)}$$

To simplify calculations, the sensor model is divided into two cases: *F* (non-zero measurements) and $\bar{F}$ (zero measurements). The expected entropy $E({\hat{H}}_{k+1}\left({\hat{z}}_{k+1}\right))$ at the time k+1 varies according to random variable ${\hat{z}}_{k+1}$ and can be expressed as follows:

(20) $$\begin{array}{cc} E({\hat{H}}_{k+1}) & =\sum_{{\hat{z}}_{k+1}=0}^{{\hat{z}}_{k+1}=1}p\left({\hat{z}}_{k+1}|\theta_{k+1}\right)*{\hat{H}}_{k+1}\left({\hat{z}}_{k+1}\right)\\ \; & =-\sum_{{\hat{z}}_{k+1}=0}^{{\hat{z}}_{k+1}=1}p\left({\hat{z}}_{k+1}|\theta_{k+1}\right)*\sum_{m=1}^{M}w_{k+1}^{\left(m\right)}\left({\hat{z}}_{k+1}\right)\log w_{k+1}^{\left(m\right)}\left({\hat{z}}_{k+1}\right) \end{array}$$

The probability $p({\hat{z}}_{k+1}|\theta_{k+1})$ can be obtained from Equation (8). The reward function of infotaxis II is then given by:

(21) $$\begin{array}{cc} I_{\mathrm{info}2\_}\left(a_{k}\right) & =E\left({\hat{H}}_{k+1}\left({\hat{z}}_{k+1}\right)\right)-H_{k}\\ \approx & \sum_{{\hat{z}}_{k+1}=0}^{{\hat{z}}_{k+1}=1}p\left({\hat{z}}_{k+1}|\theta_{k+1}\right)*\sum_{m=1}^{M}w_{k+1}^{\left(m\right)}\left({\hat{z}}_{k+1}\right)\log w_{k+1}^{\left(m\right)}\left({\hat{z}}_{k+1}\right)+\sum_{m=1}^{M}w_{k}^{\left(m\right)}\log w_{k}^{\left(m\right)} \end{array}$$

In 2018, Hutchinson et al. proposed a framework based on the maximum entropy sampling principle, referred to as entrotaxis [29]. The maximum entropy sampling principle is newly employed to guide the searcher. The approach follows a similar procedure to infotaxis II, utilizing the probabilistic representation of the source. However, the reward function considers the entropy of the predictive measurement distribution rather than that of the entropy of the expected posterior. Essentially, entrotaxis directs the searcher to locations characterized by the highest uncertainty in the next measurement, while infotaxis II moves the searcher to locations where the next measurement is expected to minimize posterior uncertainty. The reward function of entrotaxis, simplified using the sensor model and approximated using the particle filter, is expressed as follows:

(22) $$I_{entro\_}\left(a_{k}\right)=-\sum_{{\hat{z}}_{k+1}=0}^{{\hat{z}}_{k+1}=1}p\left({\hat{z}}_{k+1}|\theta_{k+1}\right)*\sum_{m=1}^{M}w_{k+1}^{\left(m\right)}\left({\hat{z}}_{k+1}\right)logw_{k+1}^{\left(m\right)}\left({\hat{z}}_{k+1}\right)$$

Using information entropy as the decision metric, the agent must effectively illustrate the advantages and disadvantages of alternative movement directions to facilitate optimal decision-making. However, during the initial stage of searching away from the source, the probability of detecting odor particles is nearly zero. The update of the source location posterior probability map relies on the gradual update of odor-free sampling particles and their probability updates through the particle filter. As the particle count is significantly lower than the number of grid cells, this discrepancy makes it challenging to accurately assess the quality of the selected movement direction. This reduction in particle count adversely effects search efficiency.

In comparison to information entropy, $R\overset{´}{e}nyi$ divergence offers broader applicability for information measurement. The information increment derived from the $R\overset{´}{e}nyi$ measure focuses on low-probability regions and allows for the differentiation of small differences between probability distributions. In contrast, the information increment derived from Shannon entropy may lead to suboptimal decisions by the agent and reduce traceability efficiency. Therefore, the $R\overset{´}{e}nyi$ divergence can be utilized as the reward function, and the $R\overset{´}{e}nyi$-infotaxis(RI) cognitive strategy can be established based on this increment. The formula is provided below:

(23) $$I_{R\overset{´}{e}nyi}\left(a_{k}\right)=\frac{1}{\alpha -1}log\int p{\left(\theta_{k}|z_{1:k}\right)}^{\alpha }p{\left(\theta_{k+1}|z_{1:k+1}\right)}^{1-\alpha }$$

In Equation (23), α is a hyperparameter, where typically α>0 and α≠1. When α=1, the $R\overset{´}{e}nyi$ divergence degenerates to the Kullback–Leibler (K-L) divergence. When α<1, the $R\overset{´}{e}nyi$ divergence becomes more sensitive to low-probability regions, while for α>1 the Rényi divergence becomes more sensitive to high-probability regions. After approximating the posterior probability using the particle filter, $I_{R\overset{´}{e}nyi\_}\left(a_{k}\right)$ is defined as follows:

(24) $$I_{R\overset{´}{e}nyi\_}\left(a_{k}\right)=\frac{1}{\alpha -1}log\sum_{m=1}^{M}{\left(w_{k}^{\left(m\right)}\right)}^{\alpha }{\left(w_{k+1}^{\left(m\right)}\right)}^{1-\alpha }$$

After dividing the sensor model into two cases, the equation becomes:

(25) $$I_{R\overset{´}{e}nyi\_}\left(a_{k}\right)=\sum_{{\hat{z}}_{k+1}=0}^{{\hat{z}}_{k+1}=1}p\left({\hat{z}}_{k+1}|\theta_{k+1}\right)\frac{1}{\alpha -1}log\sum_{m=1}^{M}{\left(w_{k}^{\left(m\right)}\left({\hat{z}}_{k+1}\right)\right)}^{\alpha }{\left(w_{k+1}^{\left(m\right)}\left({\hat{z}}_{k+1}\right)\right)}^{1-\alpha }$$

#### 4.5.2. Gravitational Potential Field

The gravitational potential field is a concept borrowed from the principle of universal gravitation in physics, describing how the attractive force between objects in a field varies with distance. In the multi-agent odor source search task, the agent and potential source term are modeled such that the potential source term generates a virtual gravitational pull on the agent, drawing it towards the expected odor source. It is considered that the magnitude of the attractive force exerted by the potential source term on the agent is a reward function that guides the agent towards the potential odor source. At large distances, the attractive force from the potential source term is small, where the attractive force increases as the distance decreases. The formula for calculating the attractiveness of movable position $a_{k}$ is as follows:

(26) $$G\left(a_{k}\right)=\sum_{m=1}^{M}\frac{w_{k}^{\left(m\right)}}{\left|\right|a_{k}-r_{o,k}^{\left(m\right)}{\left|\right|}^{\gamma }}$$

where $\left|\right|a_{k}-r_{o,k}^{\left(m\right)}\left|\right|$ denotes the distance between the agent and the *m*-th potential source term after the agent takes the action, $w_{k}^{\left(m\right)}$ represents the weight of the *m*-th potential source term, and γ controls the rate at which the attractive force decays with distance. Setting γ=0.1 addresses the issue of the attractive force becoming excessively small at large distances, while maintaining the physical significance of the gravitational field. Furthermore, the weight of the potential source term is used to adjust the influence rate of different potential sources, giving higher priority to closer and higher-probability sources.

#### 4.5.3. Gravitational-Rényi Infotaxis Congnitive Strategy

**Figure 6:**
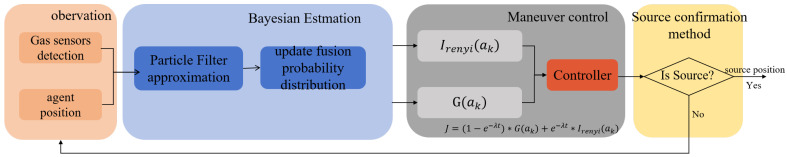
Framework of hybrid cognitive strategies.

Classical cognitive strategies are concerned with the balance between exploration and exploitation in the search process. Exploration is defined as the investigation of unknown space, whereas exploitation is defined as the use of pre-existing information. An effective cognitive strategy must maintain this balance. In infotaxis, the exploitation term is relevant only when the agent is near the odor source, whereas in infotaxis II and entrotaxis, this term is eliminated. Excessive exploration leads to a reduction in search efficiency as the number of odor detections increases. In this study, we propose a hybrid cognitive strategy named Gravitational-Rényi Infotaxis (GRInfotaxis), which integrates gravitational potential fields with Rényi divergence to achieve efficient odor source localization. In the GRInfotaxis framework, the attractive force from the gravitational potential field serves as the exploitation component, guiding agents toward probable odor source locations. Simultaneously, Rényi divergence is employed as the exploration component, encouraging agents to investigate regions that have not been adequately explored. By dynamically adjusting the weights of these two components, the proposed strategy effectively balances exploration and exploitation throughout the search process. The main framework is illustrated in Figure 6. As shown in the figure, the potential source term in particle filtering is updated using real-time measurements from sensors and prior probabilities. The updated weights and position are employed to compute the decision function, which determines the movement direction and serves as prior information for the next step. If the odor source is not located, the process continues with sensor measurements to update the potential source probability map until the odor source is found. The differences between GRInfotaxis and the classical approach are shown in Table 1.

#### 4.5.4. Cooperative Gravitational-Renyi Infotaxis Congnitive Strategy

**Figure 7:**
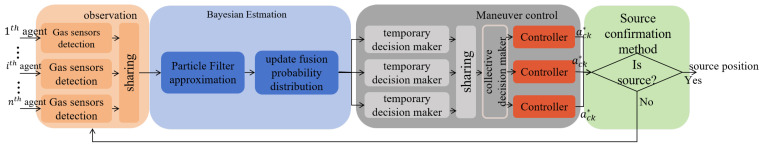
Framework of CGRInfotaxis.

In estimating and searching for odor sources using multi-agent systems, a distributed decision-making structure for coordinated cooperation through mutual information exchange among sensors is required. This study extends the hybrid cognitive strategy to the multi-agent domain, proposing a new collaborative hybrid cognitive strategy, termed Cooperative Gravitational-Rényi Information Infotaxis (CGRInfotaxis). During the multi-agent search process, multiple sensors enhance the accuracy of source estimation through the exchange of measurement values, allowing for faster and more precise estimates. During the search, each agent performs a temporary update of its posterior probability distribution based on its own measurements and those shared by neighboring agents. This update allows the agent to derive a provisional optimal decision. The provisional decisions are then shared across the team, facilitating iterative refinement of the posterior probabilities. This process is repeated iteratively until a consensus decision is achieved among all agents, ensuring coordinated and efficient source estimation. The entire process is illustrated in Figure 7.

In the process of utilizing multi-agent systems for odor source detection, the posterior probabilities are updated based on measurements from multiple real-time sensors, which are capable of sharing communication. At time *k*, if the *n*-th agent moves to position $r_{\left(k,n\right)}=\left[x_{\left(x,n\right)},y_{\left(x,n\right)}\right]$ and obtains a measurement $z_{\left(k,n\right)}$, where n∈(1,2,…N) and *N* represents the number of agents, the posterior probability update formula for the *n*-th agent is given by Equation (27):

(27) $$p\left(\theta_{\left(k,n\right)}|z_{\left(1:k,n\right)}\right)=\frac{p\left(z_{\left(1:k,n\right)}|\theta_{\left(k,n\right)}\right)p\left(\theta_{\left(k,n\right)}|z_{\left(1:k-1,n\right)}\right)}{p(z_{\left(1:k,n\right)}|z_{\left(1:k-1,n\right)})}$$

Equation (27) is derived from Equation (7), where $p(z_{\left(1:k,n\right)}|\theta_{\left(k,n\right)})$ denotes the likelihood function of the *n*-th agent at time *k*. In a multi-agent system, where the likelihood function is updated based on sensor measurements from multiple agents, it is assumed that the measurements acquired by each agent are mutually independent and solely influenced by the release of the odor source and not by other agents. Specifically, if the sensor measurements at different time steps and locations, $z_{\left(k,1\right)},z_{\left(k,2\right)},\ldots ,z_{\left(k,N\right)}$, are independent, and the likelihood function for each sensor at time *k* is $p(z_{\left(1:k,n\right)}|\theta_{\left(k,n\right)})$, then the joint likelihood function of multiple sensors can be expressed as Equation (28). Consequently, the joint posterior probability distribution for *N* agents is expressed as Equation (29), where $p(z_{k}|z_{1:k-1})$ represents the normalization constant.

(28) $$p\left(z_{\left(k,1\right)},z_{\left(k,2\right)},\ldots ,z_{\left(k,N\right)}|\theta_{\left(k,n\right)}\right)=\prod_{n=1}^{N}p\left(z_{\left(1:k,n\right)}|\theta_{\left(k,n\right)}\right)$$

(29) $$p\left(\theta_{k}|z_{1:k}\right)=\frac{\prod_{n=1}^{N}p\left(z_{\left(1:k,n\right)}|\theta_{\left(k,n\right)}\right)p\left(\theta_{k}|z_{1:k-1}\right)}{p(z_{k}|z_{1:k-1})}$$

The posterior probability is approximated through particle filtering, yielding:

(30) $${w^{\prime }}_{\left(k,n\right)}^{\left(m\right)}=\prod_{n=1}^{N}p\left(z_{\left(k,n\right)}|\theta_{\left(k,n\right)}^{\left(m\right)}\right)w_{\left(k-1,n\right)}^{\left(m\right)}$$

The normalized weights are expressed as:

(31) $$w_{\left(k,n\right)}^{\left(m\right)}=\frac{{w^{\prime }}_{\left(k,n\right)}^{\left(m\right)}}{\sum_{i=1}^{M}{w^{\prime }}_{\left(k,n\right)}^{\left(i\right)}}$$

**Figure 8:**
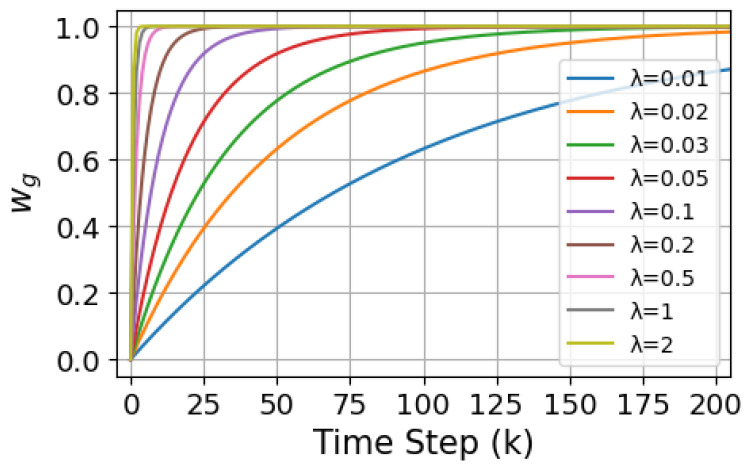
$w_{g}=1-e^{-\lambda k}$ for different λ values.

In the multi-agent odor source detection process, the potential source term exerts an attractive force on each agent. The total gravitational potential is considered as the exploitation term, with $G_{coo}$ driving multiple agents toward the potential source. The gravitational potential experienced is expressed as follows:

(32) $$G_{coo}=\sum_{n=1}^{N}g_{\left(k,n\right)}\sum_{m=1}^{M}w_{\left(k,n\right)}^{\left(m\right)}\left|\right|r_{\left(k,n\right)}-r_{o,(k,n)}^{\left(m\right)}\left|\right|$$

where $g_{\left(k,n\right)}$ represents the weight of the gravitational potential experienced by the *n*-th agent, with the condition that $g_{\left(k,n\right)}=\frac{G(a_{\left(k,n\right)})}{\sum_{n=1}^{N}G\left(a_{\left(k,n\right)}\right)}$. $g_{\left(k,n\right)}$ changes according to the magnitude of the gravitational force experienced by the agent; the larger the gravitational force, the higher the weight. $G(a_{\left(k,n\right)})$ denotes the gravitational potential acting on the *n*-th agent. The gravitational force is used as the exploitation term in the decision function, while the Rényi divergence is used as the exploration term in the reward function. To improve the adaptability of the algorithm, a dynamic weight adjustment mechanism is incorporated. A dynamic adjustment factor is introduced to regulate the strength of the gravitational force and Rényi divergence in the decision function based on time, to better balance the exploitation and exploration terms. The decision function is as follows:

(33) $$J=w_{g}*G_{coo}\left(a_{ck}\right)+w_{i}*I_{R\overset{´}{e}nyi\_}\left(a_{ck}\right)$$

where $w_{g}=1-e^{-\lambda k}$ and $w_{g}+w_{i}=1$. The weight of the attractive force changes with the time step *k*. In the early stages of the search, the agent has less useful information and therefore focuses more on exploring unknown regions, with $w_{i}>w_{g}$, prioritizing exploration. As the search progresses, the agent gathers more useful information and gradually focuses on known regions, allowing the weight of the exploitation term to increase, thus facilitating faster identification of the odor source. λ represents the balance factor, which controls the decay rate of $w_{g}$. The weights are designed to vary as an exponential function of the time step *k* through the parameter λ, enabling a dynamic transition from exploration to exploitation. Based on the system’s search time steps *k* and the expected transition between exploration and exploitation, the value of λ can be initially estimated theoretically. Under the assumption of time steps *k* = 0–200, the plotted curve of $w_{g}$ is shown in Figure 8:

From the curve, it can be observed that the value of λ has a critical impact on $w_{g}$. As *k* approaches infinity, $w_{g}$ tends towards 1. When λ is small (e.g., λ=0.01,0.02,0.03,0.05), $w_{g}$ approaches 1 quickly, leading to an early bias towards exploitation of the information already gathered.This may result in premature particle convergence during the search process, causing insufficient information collection, thereby lowering the search efficiency or causing instability. Assuming that the transition from exploration to exploitation is to be completed within the time step range k1, the following condition must be satisfied, as shown in Equation (34):

(34) $$e^{-\lambda k_{1}}\approx \epsilon$$

Then, $\lambda =\frac{ln\epsilon }{k_{1}}$. Anticipate that $w_{g}$ will rise to around 0.8 at approximately $k_{1}=100$ steps and $w_{i}$ drop to around 0.2. Therefore, $\lambda =\frac{ln\epsilon }{k_{1}}\approx 0.02$. To maintain the balance between exploration and exploitation, we set λ=0.02, which ensures a smoother dynamic adjustment of $w_{g}$, preventing over adjustment due to fluctuations in distance and maintaining the system’s stability.

However, it must be noted that the parameter λ is statically defined based on mathematical derivation, and this static parameter design offers a two-fold advantage. First, the smoothing property of the exponential function effectively mitigates parameter oscillations caused by system fluctuations, ensuring the stability of the search process. Second, the fixed-parameter configuration significantly reduces algorithmic complexity, facilitating efficient implementation and validation in a numerical simulation environment.

Nevertheless, this approach also has inherent drawbacks. Static parameters exhibit significant limitations when the environmental context changes, particularly when the simulation range varies. In this work, static parameters are adopted to preliminarily verify the theoretical efficiency and fundamental validity of the proposed CGRInfotaxis strategy through numerical simulations. Future work will focus on developing adaptive parameter tuning algorithms to further enhance the engineering applicability of the strategy.

In selecting the optimal decision, the value of *J* for each possible movement direction is calculated. The iterative process allows agents to collaboratively choose the direction that maxes the collective reward, expressed as:

(35) $$a_{ck}^{*}=\max\left(J\right)=\max\left[\left(1-e^{-\lambda k}\right)*G_{coo}+e^{-\lambda k}*I_{r\overset{´}{e}nyi\_}\right]$$

where $a_{ck}^{*}$ represents the best common movement direction of multiple agents. The agents iteratively seek the optimal movement strategy, progressing step by step until the odor source is located. If the odor source is not found within the specified maximum search time, the search is deemed unsuccessful. Algorithm 1 describes the entire process of the interactive decision making for the CGRInfotaxis.
**Algorithm 1** CGRInfotaxis for Multi-Agent System**Input:** Searching environment parameters, particle filter parameters, and agent parameters.**Output:** Action of multi-agent and the position of predicted source1:**for** $k=1,2,\ldots k_{max}$ **do**2:   **for all** agent n∈{1,2,…,N} **do**3:   **Observation**: $z_{k,n}$ read multi-agent measurements;4:   **Share Sensor Measurements:** Broadcast $z_{k,n}$ to all agents and receive $z_{k}=\left\{z_{k,1},z_{k,2},\ldots ,z_{k,N}\right\}$;5:   **end for**6:   $w_{\left(k,n\right)}^{\left(m\right)}\leftarrow w_{\left(k-1,n\right)}^{\left(m\right)}$ using Equations (30) and (31);7:   **if** $M_{eff}<M_{th}$ **then**8:   Resampling and set $w_{k}^{\left(m\right)}=\frac{1}{M}$;9:   **end if**10: **Compute Initial Temporary Decisions for all Agents**11: **while** not Decisions reach consensus **do**12:    **for all** agent n∈{1,2,…,N} **do**13:     **Decision Sharing:** Broadcast temporary decision and receive other agent decisions14:     **for all** $a_{k}\in A_{k}$ **do**15:      $p({\hat{z}}_{k+1}|\theta_{k+1})$ for other agent decisions and $a_{k}$ using Equation (29)16:      $w_{\left(k+1,n\right)}^{\left(m\right)}$ using Equation (30);17:      $I_{R\overset{´}{e}nyi\_}\left(a_{ck}\right)$ computation using Equation (25);18:      $G(a_{ck})$ computation using Equation (26);19:      $J(a_{ck})$ computation using Equation (33);20:     **end for**21:     $a_{\left(ck,n\right)}^{*}=\max\left(J\left(a_{ck}\right)\right)=\max\left[\left(1-e^{-\lambda k}\right)*G_{coo}+e^{-\lambda k}*I_{r\overset{´}{e}nyi\_}\right]$ select the motion control;22:     Update the decision of agent n23:    **end for**24:    **Check consensus status:**25:    **if** Decisions reach consensus **then**26:     **output the action of multi-agent**27:     $\left[x_{k+1},y_{k+1}\right]$ of every agent computation using Equation (14)28:    **end if**29:   **end while**30:   **if** source confirmation conditions reached **then**31:     **output the position of predicted source**32:     **break**33:    **end if**34:**end for**

## 5. Numerical Simulations

A numerical simulation is designed to verify the performance of the proposed CGRInfotaxis. First, we determine the value of the α in the Rényi divergence. We then provide illustrative runs of source search and estimation using a multi-agent. Monte Carlo simulation results are then presented to compare the performance between different approaches.

### 5.1. The Value of α

α is an important parameter in the calculation of Rényi divergence and is used to measure the difference between two probability distributions. The choice of α also affects how agents explore the unknown environment. As described in Section 4.5.1, alpha controls the sensitivity of Rényi divergence to high-probability and low-probability regions. To obtain the optimal range of α for CGRInfotaxis, we randomly selected four alpha values (0.4, 0.8, 2, and 3) and 100 odor sources with varying positions and release intensities. The experiments were repeated with different α values, and the results are shown in Table 2:

The search performace is evaluated with four metrics: Success Rate (SR), Mean Search Time (MST), Mean Error (ME), and Standard Deviation (STD). From the experimental data, it can be observed that different alpha values maintain a relatively high SR. However, when α<1, the MST is significantly lower than when α>1. This may be because, for α>1, agents tend to repeatedly validate local high-probability regions and, especially in complex environments, this behavior significantly extends the search time. Additionally, larger α values also show slightly higher ME, which may be due to agents’ excessive reliance on local high-probability regions, resulting in a reduced global coverage capability.

The change in STD indicates that as α increases, the stability of the search results decreases, exhibiting greater volatility across different experiments. In contrast, smaller alpha values make agents more sensitive to low-probability regions, encouraging them to prioritize exploring uncovered areas, which is suitable for expanding the search range in the early stages of the search. However, this behavior may result in the neglect of local high-probability regions, slightly reducing the success rate. Larger alpha values, on the other hand, tend to focus more on exploiting high-probability areas, but this also increases the search time and may lead to greater volatility in the search results.

**Figure 9:**
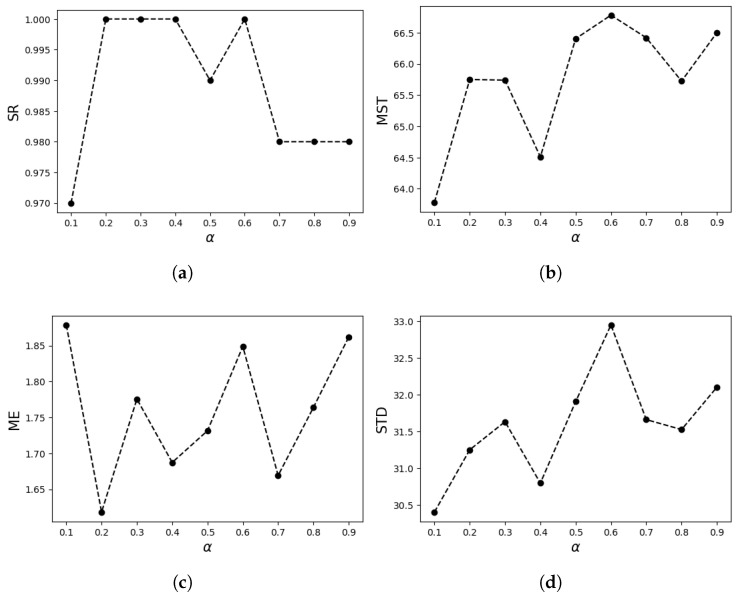
Performace of α in (0, 1). (**a**) SR. (**b**) MST. (**c**) ME. (**d**) STD.

In order to study the influence of alpha value on search results when α<1, uniform values were selected from the range (0, 1) to obtain 9 different α values. One hundred random odor sources were randomly selected to conduct experiments on 9 α values, and the results are as shown in Figure 9. Experimental results show that when α is determined between 0.1 and 0.9, the SR of the system is maintained at a high and stable level, while performance indicators such as MST, ME, and STD do not exhibit significant variations with changes in the alpha parameters and remain in a relatively ideal state under different alpha values. Therefore, to increase the diversity of exploration in the experiment, α values are uniformly distributed within the range [0.1, 0.9], i.e., α=U(0.1,0.9), which does not cause significant performance degradation. In addition, the introduction of certain randomness also increases the robustness of the algorithm, prevents it from over-relying on a specific reward mechanism, and helps the algorithm escape from local optima to a certain extent, improving the overall adaptability of the system.

### 5.2. Illustrative Run

To show the characteristics of CGRInfotaxis, we perform illustrative simulation runs with three agents. The simulation is designed in a two-dimensional space, and the particle filter framework is used. Every algorithm follows the same simulation settings while the average consensus iteration number is 100. The units in the simulation are arbitrary units (a.u.) adopted from previous similar studies [9,17,28,29]. The simulation parameters are given as:

- True source term estimation parameters: the source location $r_{s}=\left[10,50\right]$, the start location $r_{a}=\left(\left[180,50\right],\left[180,60\right],\left[180,40\right]\right)$, and the source releasr strength $Q_{s}=1$;
- Searching environment parameters: search area Ω=200∗100, isotropic diffusivity D=1, mean wind speed V=1, and the lifetime of emitted source particle τ=400;
- Particle filter parameters: the number of particles N=50∗50;
- Agent parameters: sensing size a=1, sensing time Δ=1s, feasible control decision A=[↑,↓,←,→,·]

**Figure 10:**
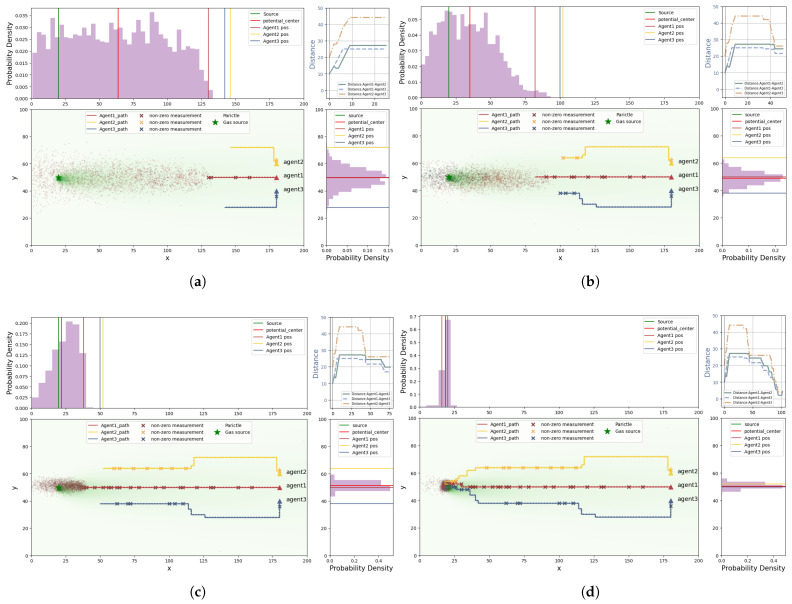
Illustrated run of CGRInfotaxis. (**a**) k=26. (**b**) k=52. (**c**) k=77. (**d**) k=103.

The CGRInfotaxis method illustrated in Figure 10 demonstrates the process of odor source searching by three agents. The reddish-brown trajectory represents the first agent, the yellow trajectory represents the second agent, and the blue trajectory represents the third agent. The true odor source is indicated by a green pentagram, while the green wisps in the figure represent the diffused odor plumes. The particles from the particle filter are represented by reddish-brown gradient particles, with darker colors indicating greater weights. In the histogram, the green vertical line indicates the true position of the odor source, the red vertical line indicates the center position of the particles, and the remaining three vertical lines, each in different colors, represent the current positions of the three agents. In the top right corner, the line graph represents the distance between the three agents as it changes with search time, with distinct colors and line styles to differentiate the agents. The entire search process is provided in Supplementary Material.

**Figure 11:**
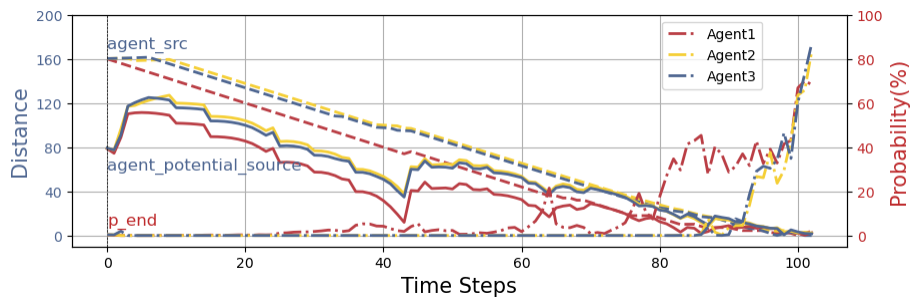
Performace of distance and probability.

From the figure, it can be seen that the three agents, starting from their initial locations, begin with a high weight on the exploration term in the early search phase. The agents initially perform a decentralized search to cover a larger area, moving in three different directions to explore unknown regions and gather more useful information. This also prevents the agents from overlapping and searching the same area. As the search progresses, the agents reach an equilibrium, each occupying a distinct search area and maintaining a proper distance to improve coverage efficiency. The particles also move from a uniform distribution to an unknown region, where the number of particles in the known region is gradually decreasing. At this time, the algorithm’s weight has not yet shifted towards exploitation. As the agents gradually sense odor source information, they converge toward the target odor source through collaboration and information sharing. The distance between agents gradually decreases, and the weight of the exploitation term increases, guiding the agents toward the odor source. The particles also converge rapidly towards the location of the odor source. In the later stage of the search, the rate of decrease in the distance between agents accelerates, which is due to the increasing weight of the exploitation term. In the final stage of the search, the agents can be seen wandering around the odor source, and the distance between them slightly increases. The particles eventually converge near the odor source, forming a distinct peak around the source. Ultimately, the agents cease movement, determining the final odor source location.To better analyze the entire search process, the variations in distance and probability throughout the search process are depicted in Figure 11.

The line graph depicts the distances between the agents and the real odor source and the distances between the agents and the potential source terms, as well as the probability of finding the source. Different colors represent different agents: red represents agent 1, yellow represents agent 2, and blue represents agent 3, as in Figure 11. In the graph, “- -” represents the distance between the agent and the real odor source, “–” represents the distance between the agent and the potential source term, and “-.” represents the probability of the agent finding the source. From the line graph, it can be observed that, in the early search phase, the distances between agent 2 and agent 3 and the real odor source increase slightly. This is because, in the early stage, the weight of the exploration term is higher, and the agents initially disperse to search, resulting in a slight increase in distance. As the search progresses, the distances between the agents and the real odor source gradually decrease with the increasing search time, demonstrating that the agents are moving toward the odor source. This indicates that our proposed algorithm effectively guides the agents’ search.

From the line graph of the distance between the agents and the potential source term, it is evident that the trend of change is similar to that of the real odor source, indicating that the prediction of the potential source term has high accuracy. In the early stage of the search, the distance between the agent and the potential source term increases, which can be explained by two factors: on one hand, due to the higher weight of the exploration term initially, the agents perform a dispersed search, causing the distance between the agent and the potential source term to temporarily increase; on the other hand, due to the dynamic changes of the potential source term being more flexible, and the agent’s movement speed being limited by a fixed step size, it is difficult for the agent to respond quickly to the rapid changes in the potential source term.

At the initial stage of the search, the probability of detecting the odor source for the agents is always zero. This phenomenon is mainly attributed to the agents continuously following the positions of the high-probability potential source terms, and since the agent’s step size is fixed the agent’s movement speed is lower than that of the potential source term, causing the probability of surrounding potential source terms to gradually approach zero. As the distance between the agent and the potential source term gradually decreases, the probability of detecting the odor source begins to show significant fluctuations. As the distance further converges, the probability of detecting the odor source increases significantly and approaches 100%. This result shows that the agents’ certainty about the location of the real odor source gradually increases.

Throughout the entire search process, it can be observed that the probability of finding the odor source for agent 1 increases first. However, after reaching a certain value, the curve becomes relatively stable with minor fluctuations. Meanwhile, the probabilities for agent 2 and agent 3 also rapidly increase and move toward the position of agent 1. This phenomenon reflects the information exchange and collaborative search process between the agents. Agent 1 approaches the odor source first; subsequently, its behavior guides the other agents towards the real odor source, ultimately achieving the collaborative search task and pinpointing the real odor source.

**Figure 12:**
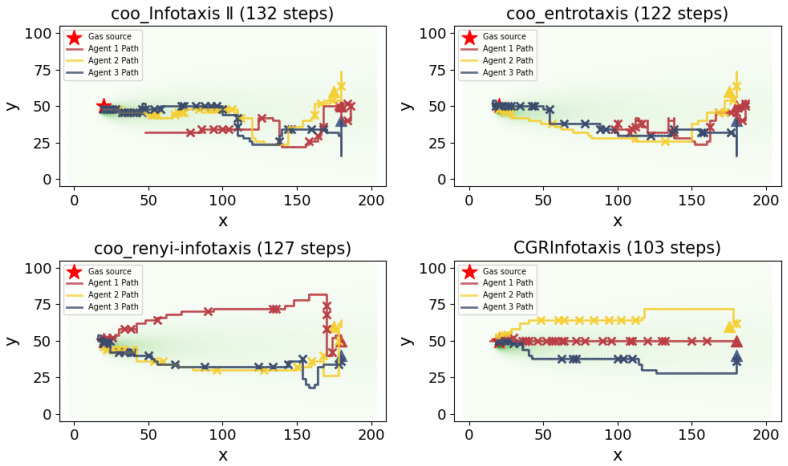
Comparison of different algorithms in multi-agent systems.

In order to compare the differences between various cognitive strategies, we simulated four different algorithms using identical parameters, including coo_infotaxis II, coo_entrotaxis, and coo_Rényi-infotaxis, and compared the results with the proposed CGRInfotaxis by plotting the corresponding search path maps. As shown in Figure 12, all agents begin from the same initial position. The four strategies were used to search for the odor source. It can be seen that the proposed CGRInfotaxis locates the odor source in the shortest time, demonstrating a clear advantage in convergence speed, which allows it to find the odor source more rapidly. This advantage is primarily attributed to the effectiveness of the information sharing strategy between agents and the dynamic balance between the exploration and exploitation terms. The agents’ paths show minimal overlap, efficiently guiding them to initially explore diverse regions before converging to the odor source based on shared information. In contrast, the paths of the agents in the other three algorithms exhibit considerable overlap, especially as they approach the odor source. This indicates poor collaboration among the agents, leading to significant resource waste and a decrease in overall search efficiency. The complete search process for the four strategies is provided in the Supplementary Materials.

### 5.3. Monte Carlo Simulations

To validate the accuracy and effectiveness of the proposed algorithm, we conducted Monte Carlo simulation experiments comparing cognitive strategies based on multi-agent systems. First, we fixed the odor source location and performed 50 iterations using the simulation parameters from Section 5.2 to enhance the accuracy and reliability of the results, identifying and eliminating random errors. To assess the robustness of the algorithm, we performed Monte Carlo simulations at 100 different random odor source locations, with the release rates of different odor sources set as random numbers between 1 and 10, while keeping the other simulation parameters the same as those in Section 5.2. Finally, we evaluate the effect of the number of agents on cognitive strategies.

#### 5.3.1. Comparison Simulations with Fixed Odor Source Locations

**Figure 13:**
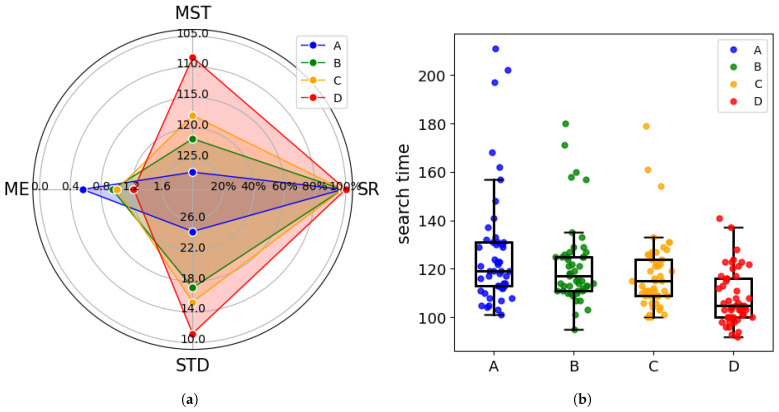
Performance comparison of 50 search results (A = cooperative infotaxis II, B = cooperative entrotaxis, C = cooperative Rényi-infotaxis, D = CGRInfotaxis). (**a**) Radar map of different strategies. (**b**) Search time distribution.

When the odor source location remains unchanged, we conduct multiple measurements to avoid the influence of experimental randomness and the stochastic nature of the process on the algorithm. To better evaluate the algorithm’s performance, we use three evaluation metrics: search Success Rate (SR), Mean Search Time (MST), Mean Error (ME), and Standard Deviation (STD) of search time to compare the algorithms. Table 3 and Figure 13 provide a comparison of the performance of different algorithms.

Table 3 presents the success rate, mean search time, mean error, and standard deviation of search times for different stratiegies. To facilitate clearer comparison, Figure 13 shows a radar map visualizing the evaluation metrics along with the search time distribution across 50 trials. It can be observed that in the 50 repeated experiments, all four cognitive strategies demonstrated high success rates, with the CGRInfotaxis strategy achieving a perfect success rate of 100%. In terms of average search time, the CGRInfotaxis strategy outperformed the others significantly, reducing the average search time by 14.7% compared to coo_infotaxis II and by 10.9% compared to coo_entrotaxis.

Figure 13b illustrates the distribution of search times for the four strategies. The CGRInfotaxis strategy exhibits the lowest median search time, with a more concentrated distribution and fewer outliers. To precisely assess the consistency and stability of each strategy, we quantified the standard deviation of search time over 50 trials, as shown in Table 3 and Figure 13a. The CGRInfotaxis strategy exhibits the lowest standard deviation, indicating the most consistent results. In contrast, the coo_infotaxisII strategy shows the highest standard deviation, reflecting greater fluctuations across experiments, as indicated by the number of outliers in Figure 13b.

Regarding mean error, the CGRInfotaxis algorithm performed slightly worse, but the error remained below 2. In the radar map, higher values in each dimension correspond to better performance in that aspect. From Figure 13a, it is clear that the CGRInfotaxis strategy excels in SR, MST, and STD, whereas the coo_infotaxisII strategy outperforms in search accuracy. Therefore, our strategy significantly enhances search efficiency and stability, albeit at the cost of accuracy. Taking into account the SR, MST, ME, and STD, the CGRInfotaxis strategy performs exceptionally well, occupying a larger area in the radar chart, which makes it ideal for scenarios demanding high efficiency and stability.

#### 5.3.2. Comparison Simulations with Random Odor Source

**Figure 14:**
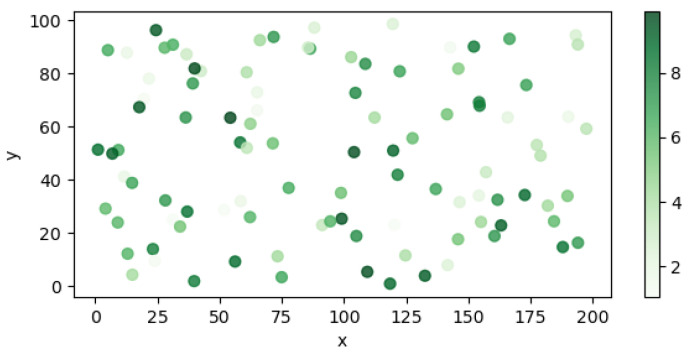
Randomly distributed odor sources.

The multi-agent odor source localization experiment is simulated with three agents, with initial search points uniformly distributed in the downwind area at specific coordinates of [(180, 50), (175, 60), (180, 40)]. In a simulated environment of size 200∗100, 100 random odor sources are selected. The odor source release intensity, denoted as $Q_{s}$, is randomly assigned a value between 1 and 10. The distribution of odor source positions is illustrated in Figure 14, where the shading intensity represents the magnitude of the release intensity $Q_{s}$.

**Figure 15:**
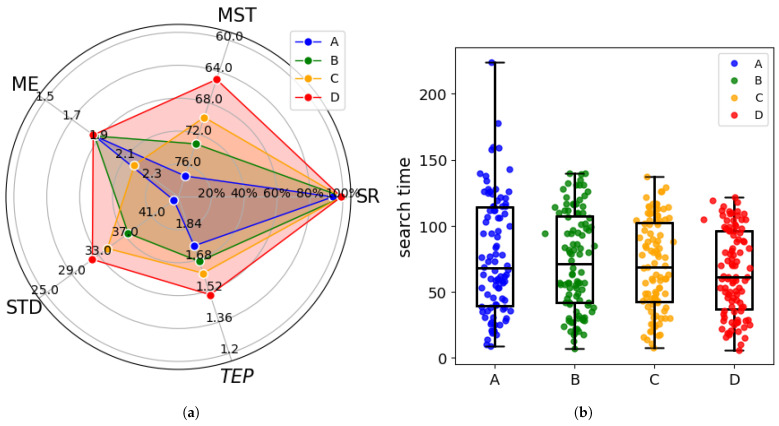
Performance comparison of 100 random odor sources (A = cooperative infotaxis II, B = cooperative entrotaxis, C = cooperative Rényi-infotaxis, D = CGRInfotaxis). (**a**) Radar map of different strategies. (**b**) Search time distribution.

Each cognitive strategy is simulated 100 times using the parameters detailed in Section 5.2. We performed Monte Carlo simulations comparing the multi-agent algorithms cooperative infotaxis, cooperative entrotaxis, cooperative Rényi-infotaxis, and cooperative GRInfotaxis using the same parameters. The same as in Section 5.2, we compare the algorithms based on SR, MST, ME, and STD of search time. To compare path redundancy between strategies, a metric called Path Efficiency (PE) is introduced, as defined in Equation (36). This metric quantifies the ratio of the actual distance traveled by the agents to the shortest possible distance, thereby measuring the deviation of the agents’ actual paths from the shortest paths during odor source localization. A lower path efficiency indicates that the agents’ actual paths are closer to the shortest paths. In multi-agent systems, Team Path Efficiency (TPE) is calculated by defining the shortest path as the average of the shortest paths of all agents, as shown in Equation (37), to quantify the overall path redundancy across the team.

(36) $$PE=\frac{d_{actual}}{d_{shortest}}$$

(37) $$TPE=\frac{d_{actual}}{d_{ave_{s}hortest}}$$

where $d_{actual}$ represents the distance traveled by the agent, $d_{shortest}$ represents the shortest distance, and $d_{ave_{s}hortest}$ represents the average of the shortest paths. TPE reflects the degree of path optimization and the strength of exploration ability of the intelligences. If the ratio is close to 1, it indicates that the path almost coincides with the shortest path. If the ratio is greater than 1, it indicates that the intelligent body has traveled a redundant path. In the design of cognitive strategies, exploratory behavior leads to path extension. Table 4 and Figure 15 show the comparative results of 100 Monte Carlo simulations.

The SR, MST, ME, STD, and TPE for the four cognitive strategies are summarized in Table 4. Figure 15 illustrates these evaluation metrics and presents the distribution of search times across 100 trials. The radar plot indicates that CGRInfotaxis performs well in MST, STD, and TPE, demonstrating high efficiency and stability. Although CGRInfotaxis is not the best-performing algorithm in terms of success rate, its performance closely approximates that of the optimal strategy. Regarding average error, differences among the four strategies are relatively minor; however, CGRInfotaxis still achieves slightly better performance. Figure 15b further illustrates the search time distribution across 100 trials. This figure clearly demonstrates that CGRInfotaxis exhibits a more centralized search time distribution and a shorter box plot. However, the search time distributions of the four cognitive strategies are more dispersed compared to those in Figure 14. This variability arises from the random selection of odor source locations. When the odor source is within the agents’ field of view, fewer steps are required; conversely, more steps are needed when the source is farther away. This is a typical outcome. The smaller standard deviation indicates that, regardless of the odor source location, the variation in the number of search steps is minimal, suggesting that CGRInfotaxis performs consistently well under different conditions. Its cognitive and collaboration strategies exhibit strong stability and consistency, validating the robustness of the cognitive approach.

#### 5.3.3. Comparison Simulations with Different Agent Numbers

**Figure 16:**
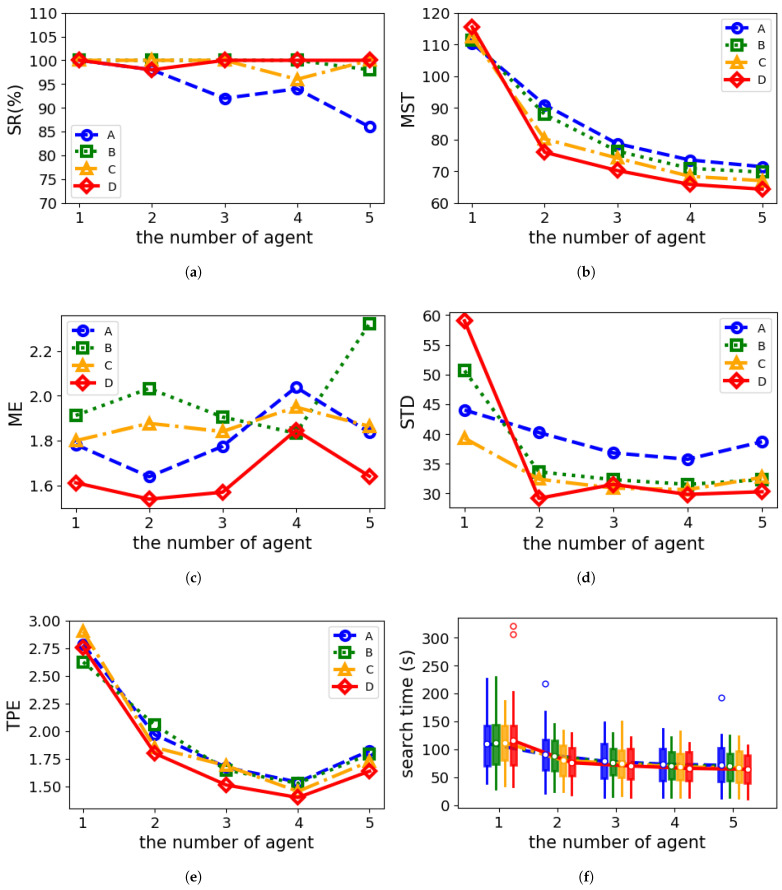
Performance comparison for different strategies (A = cooperative infotaxis II, B = cooperative entrotaxis, C = cooperative Rényi-infotaxis, D = CGRInfotaxis). (**a**) SR. (**b**) MST. (**c**) ME. (**d**) STD. (**e**) TPE. (**f**) search time.

To evaluate the impact of the number of agents on cognitive strategies, we conducted experiments using 50 randomly placed odor sources. The initial search positions of the agents are configured as shown in Table 5, following the principle of ensuring that agents are as evenly distributed as possible downwind of the odor source. Table 6 and Figure 16 present a comparative analysis of the average performance of different cognitive strategies under varying agent counts.

From Figure 16a, it can be observed that, among the four strategies, the success rates of coo_entrotaxis, coo_Rényi-infotaxis, and CGRInfotaxis remain consistently at 100%, while the success rate of coo_infotaxis II decreases as the number of agents increases, indicating its clear limitation in multi-agent cooperative search scenarios. Correspondingly, Figure 16b shows that the Mean Search Time (MST) for all four strategies significantly decreases as the number of agents increases. CGRInfotaxis exhibits particularly strong performance—except for in the single-agent scenario, its MST remains consistently lower than that of the other three strategies, highlighting a notable advantage.

Figure 16c shows the trend of the mean error across the four strategies as the number of agents increases. Although fluctuations are observed, the ME of CGRInfotaxis remains the lowest overall, further confirming its performance advantage. The STD reflects the consistency of the search outcomes across different environments. As illustrated in Figure 16d, when the number of agents is greater than one, CGRInfotaxis consistently maintains a low STD, indicating strong stability. When combined with the search time distribution in Figure 16f, it is evident that CGRInfotaxis achieves the shortest box length, signifying lower variability in search time. The slightly higher STD observed in the single-agent case is directly attributable to two outliers, as shown in Figure 16f.

Figure 16e illustrates the variation of TPE with the number of agents across different strategies. The TPE does not continuously decrease with an increasing number of agents; instead, it may increase when the number of agents becomes too large. This phenomenon aligns with the results in Figure 16b: beyond a certain point, the decrease in average search time slows significantly. Both observations highlight a core issue—within a limited search space, an excessive number of agents can lead to redundant searches. Specifically, when the number of agents surpasses a reasonable threshold, the overlap in search paths increases, resulting in a longer cumulative path length and consequently higher TPE. Simultaneously, redundant searches consume system resources, reducing the efficiency of newly added agents in covering unexplored areas and diminishing the overall improvement in average search time.

In summary, CGRInfotaxis demonstrates superior performance across several key metrics compared to other strategies, which may perform well in specific configurations but exhibit clear limitations in terms of stability and efficiency within multi-agent systems. The proposed CGRInfotaxis strategy is particularly well-suited for collaborative search scenarios involving multiple agents. In practical applications, these advantages enable the CGRInfotaxis algorithm to complete search tasks more efficiently, enhance the accuracy and stability of task execution, reduce system resource consumption, and provide more reliable technical support for multi-agent collaborative search practices in related fields.

## 6. Conclusions and Future Work

In this paper, we presented a distributed hybrid architecture for atmospheric pollution tracking, which enhances multi-agent collaborative search capabilities for odor source localization in turbulent environments, specifically improving environmental monitoring efficiency through hybrid cognitive strategies. The proposed method integrates gravitational potential fields and Rényi divergence to iteratively guide each agent toward its locally optimal position relative to other agents, with repeated updates ultimately leading to a globally optimal collective decision. Through dynamic information sharing and decision updates, agents collaboratively refine their search strategies, enhancing both efficiency and accuracy. Additionally, an adaptive balancing factor λ is incorporated to dynamically regulate the trade-off between exploration and exploitation, optimizing search performance across varying conditions. To illustrate the characteristics of the proposed strategy, an exemplary scenario is provided through numerical simulations, followed by extensive Monte Carlo studies that demonstrate the key advantages of the interactive decision optimization-based hybrid cognitive strategy over conventional methods. The results indicate that CGRInfotaxis consistently achieves higher search efficiency, lower localization error, greater success rates, and improved stability. Notably, its performance is shown to scale effectively with an increasing number of agents, reinforcing its applicability to large-scale search operations. Future research directions include extending the proposed framework to enhance robustness against robotic failures and adapting it to environments with obstacles. Furthermore, transitioning from two-dimensional to three-dimensional search spaces presents an intriguing challenge that could further advance the applicability of the method to real-world odor source localization tasks.

## Figures and Tables

**Table 1:** Comparison of four algorithms.

Method	Decision Function	Core Objective	Search Characteristics
Infotaxis II	Shannon entropy reduction in Equation (21)	Maximum entropy reduction	Delete the exploitation term and focus on local exploration
Entrotaxis	Shannon entropy in Equation (22)	Maximize Shannon entropy	Global exploration to avoid premature convergence
Rényi-infotaxis	Rényi divergence in Equation (24)	Maximize Rényi divergence	Focus on small differences between probability distributions
Gravitational-Rényi Infotaxis	Coupling Rényi divergence and gravitational potential field in Equations (25) and (26)	Maximized coupling function	Balanced exploitation and exploration, global guidance and local acceleration

**Table 2:** Performance for different α.

α	SR	MST	ME	STD
0.4	0.99	65.51	1.77	31.24
0.8	0.99	67.11	1.77	32.42
2	1.0	96.44	2.12	42.96
3	1.0	101.64	2.16	47.57

**Table 3:** Performance comparison for different methods with fixed sources (A = cooperative infotaxis II, B = cooperative entrotaxis, C = cooperative Rényi-infotaxis, D = CGRInfotaxis, bold values indicate optimal results for corresponding metrics).

	A	B	C	D
SR (%)	98	98	98	**100**
MST	127.18	121.73	1170.92	**108.44**
ME	**0.56**	0.96	1.01	1.23
STD	24.51	17.15	15.30	**11.18**

**Table 4:** Performance comparison for different methods with random sources (A = cooperative infotaxis II, B = cooperative entrotaxis, C = cooperative Rényi-infotaxis, D = CGRInfotaxis, bold values indicate optimal results for corresponding metrics).

	A	B	C	D
SR (%)	94	**100**	99	99
MST	77.33	73.22	69.83	**64.99**
ME	1.87	1.87	2.17	**1.86**
STD	44.32	37.46	34.34	**32.06**
TPE	1.75	1.67	1.61	**1.50**

**Table 5:** Agent initial positions for different team sizes.

Number	Start_pos
1	(180, 50)
2	(180, 45), (180, 55)
3	(180, 60), (180, 50), (180, 40)
4	(180, 65), (180, 55), (180, 45), (180, 35)
5	(180, 70), (180, 60), (180, 50), (18, 40), (180, 30)

**Table 6:** Performance comparison for different numbers of agents.

Number	1	2	3	4	5
**coo_infotaxis II**					
SR	100	98	92	94	86
MST	110.36	90.95	78.70	73.55	71.42
ME	1.78	1.63	1.77	2.04	1.84
STD	44.00	10.24	36.80	35.74	38.68
TPE	2.78	1.97	1.67	1.54	1.82
**coo_entrotaxis**					
SR	100	100	100	100	98
MST	111.48	87.92	76.36	70.9	69.71
ME	1.91	2.03	1.90	1.83	2.32
STD	50.64	33.62	32.29	31.50	32.28
TPE	2.62	2.05	1.65	1.53	1.79
**coo_Rényi-infotaxis**					
SR	100	100	100	96	100
MST	112.34	80.18	74.16	68.40	67
ME	1.80	1.88	1.84	1.95	1.86
STD	39.29	32.38	30.87	30.62	32.67
TPE	2.90	1.85	1.69	1.45	1.72
**CGRInfotaxis**					
SR	100	98	100	100	100
MST	115.54	75.98	70.22	65.86	64.32
ME	1.61	1.53	1.57	2.15	1.64
STD	59.03	29.14	31.49	29.81	30.26
TPE	2.76	1.80	1.51	1.40	1.64

## Data Availability

The raw data supporting the conclusions of this article will be made available by the authors on request.

## Supplementary Materials

The following supporting information can be downloaded at: https://www.mdpi.com/article/10.3390/e27080826/s1, Vidio S1: The search process of cooperative infotaxis II; Vidio S2: The search process of cooperative entrotaxis; Vidio S3: The search process of cooperative Rényi-infotaxis; Vidio S4: The search process of CGRInfotaxis.
